# Substance Dependence Among Bipolar, Unipolar Depression and Psychotic Homeless: A Canadian National Study

**DOI:** 10.3389/fpsyt.2018.00701

**Published:** 2018-12-18

**Authors:** Angelo G. I. Maremmani, Silvia Bacciardi, Julian M. Somers, Mohammadali Nikoo, Christian Schütz, Kerry L. Jang, Michael Krausz

**Affiliations:** ^1^Association for the Application of Neuroscientific Knowledge to Social Aims (AU-CNS), Pietrasanta, Lucca, Italy; ^2^Local Health Unit (Versilia Zone), Department of Psychiatry, North-Western Tuscany Region, Viareggio, Italy; ^3^“Vincent P. Dole” Dual Diagnosis Unit, Department of Clinical and Experimental Medicine, University of Pisa, Pisa, Italy; ^4^Somers Research Group, Faculty of Health Science, Simon Fraser University, Barnaby, BC, Canada; ^5^Department of Psychiatry, Institute of Mental Health, University of British Columbia, Vancouver, BC, Canada; ^6^Centre for Health Evaluation and Outcome Sciences, St. Paul's Hospital, Vancouver, BC, Canada; ^7^School of Population and Public Health, University of British Columbia, Vancouver, BC, Canada

**Keywords:** substance dependence, alcohol dependence, homeless, bipolar disorder, unipolar depression, psychotic disorder

## Abstract

**Introduction:** Homeless individuals are often mischaracterized as members of a homogeneous population that suffers from a wide mental health and addiction issues, with little consideration of potentially important differences within or between samples. The aim of the present study was to investigate the comorbidy of alcohol and/or substance dependence (ASD) and major psychiatric diagnoses (bipolar disorder, unipolar depression, and psychotic disorder) in a large Canadian sample of homeless individuals, and to examine potential sources of variability including location and ethnicity.

**Materials and Methods:** A sample of 1,585 homeless individuals were assessed for alcohol and/or substance dependence and bipolar disorder, unipolar depression and psychotic disorder with the Mini-International Neuropsychiatric Interview (version 6.0). Regional and ethnic differences in major psychiatric diagnoses between homeless with and without ASD were examined using univariate (i.e., chi-square) and multivariate (i.e., logistic regression) statistics.

**Results:** Members of the sample with ASD were found to be younger, Aboriginal, less well-educated, and born in the Americas. They were more significantly more prevalent in Western Canada and less prevalent in Central and Eastern Canada. The odds of having ASD were higher among people affected by bipolar disorder and (to a less extent) unipolar depression.

**Limitations:** Data collected were self-reported and no urinalyses were performed. We considered diagnosis of ASD according to the previous 12 months only.

**Conclusions:** Homeless people with major mental illness are at high risk for concurrent ASD, however the prevalence of ASD varies significantly between cities, and based on ethnicity and specific psychiatric diagnosis (with greater prevalence in individuals affected by bipolar disorder and, to a less extent, unipolar depression). Clinicians, administrators and policy makers should develop and deliver services based on careful assessment of the local population.

## Introduction

The comorbidity of psychiatric disorders and substance use in general populations is well-documented (1–4). In particular, individuals with schizophrenia have high rates of comorbidity involving alcohol and substance abuse (5–8) or substance misuse (9). Similarly, people with mood disorders have a high prevalence of alcohol and drug dependence (10–13). The lifetime prevalence for alcohol and drug dependence among people with bipolar disorder is over 50% (14, 15).

Research focused on homeless people has found a high prevalence of substance abuse and dependence (16–19) and serious mental illness (20–23). Unlike general population samples, homeless populations live in stressful environments, are extremely vulnerable, and are underserved by health care services (21). It may therefore be expected that the prevalence of comorbidity among the homeless may differ from the level observed in the general population (24–26). Moreover, there may be important regional and ethnic differences that have not been estimated before. Sociodemographic and regional differences are important to document because they have implications for national and local policy initiatives involving the design of housing and treatment options. However, in the absence of relevant empirical evidence, homeless people are likely to be characterized as members of a homogeneous population, leading to policies that may be ineffective for patients in a particular setting.

The present study examined the relationship between alcohol and/or substance dependence (ASD) and major psychiatric diagnosis (bipolar disorder, unipolar depression, and psychotic disorder) in a national sample of homeless people (27). Participants were recruited from five centers across Canada, including the country's three largest cities and representing highly diverse regions and ethno-racial groups. The value of a national sample is that it permits the examination of regional differences and ethnicity in the relationship between mental illness and substance use.

## Materials and Methods

### Sample

Data for the present study were drawn from the At Home/Chez Soi study that recruited participants meeting criteria for homelessness and mental illness in five cities across Canada: Vancouver, British Columbia; Winnipeg, Manitoba; Toronto, Ontario; Montreal, Quebec; and Moncton, New Brunswick. Planning for the At Home/Che Soi Study began in the spring of 2008 with the first participants recruited in the autumn of 2009 and completed in the spring of 2013. Homelessness was defined as having no fixed place to stay for more than seven nights and little likelihood of obtaining accommodation in the upcoming month, or being discharged from an institution, prison, jail or hospital with no fixed address. Precarious housing referred to people whose primary residence was a Single Room Occupancy rooming house (SRO), or hotel/motel. Precariously housed individuals were eligible for inclusion if they had two or more episodes of absolute homelessness, as defined above, in the past year. The mental disorder criterion was assessed using the Mini-International Neuropsychiatric Interview (MINI). For the current analysis we further restricted inclusion to participants who met criteria for bipolar disorder (BD), unipolar depression (UD) and psychotic disorder (PD) were selected (see below).

Strategies to ensure adequate participation included seeking referrals from a wide variety of community agencies that serve the homeless, such as shelters, drop-in centers, outreach teams, mental health teams, inpatient programs and criminal justice programs. Brochures describing the study and the eligibility criteria were widely distributed and local service providers provided advice about recruitment settings and procedures. Participants were followed for at least 2 years after enrolment. Follow-up interviews were conducted face-to-face and by telephone. For more details see Georing et al. (27). Of 611 individual excluded from the study because not eligible for the assessment, 107 individuals declined to participate (28).

### Instruments

#### Mini-International Neuropsychiatric Interview (MINI)

Lifetime and current mental and substance use disorders were estimated using the MINI International Neuropsychiatric Interview, version 6.0.0 (29). The MINI is a structured clinical interview based on Diagnostic and Statistical Manual of Mental Disorders, 4th edition (DSM-IV) diagnostic criteria (30) and the International Statistical Classification of Diseases and Related Health Problems, Tenth Revision (ICD-10) (31). The MINI has demonstrated reliability and validity (29).

#### Demographics, Service and Housing History (DSHH)

Demographic information used was age, marital status, housing situation, education, source of income, social contacts, age of first homelessness, and total amount of time being homeless. Participants were also asked whether they had ever been in prison, jail, or juvenile detention overnight or longer; and to identify which ethnic group/descent they belonged to: European/Caucasian, Aboriginal, African, Asian, Hispanic/Latin American, and Other.

Ethics board approval for the At-Home/Chez Soi Study research was obtained from 11 partnering institutions. A safety and adverse events committee was in place. The study is registered with the International Standard Randomized Control Trial Number Register and assigned ISRCTN42520374.

#### Alcohol and Substances Dependence

Alcohol and substance dependence in the previous 12 months was assessed using MINI. The MINI is sensitive to alcohol dependence, but does not distinguish between cocaine, inhalant, stimulant, opioid, tranquilizer, or hallucinogen dependence. As such, two groups were formed: homeless affected by alcohol and/or substance dependence (ASD) and homeless without any alcohol and/or substance dependence (not-ASD).

#### Psychiatric Diagnosis

Lifetime psychiatric diagnosis was estimated at baseline interview on the basis of (1) Current or past depressive episodes, (2) Current or past manic or hypomanic episodes and (3) Current or past psychotic episodes to identify subjects affected by bipolar disorder (BD), unipolar depression (UD) and psychotic disorder (PD). With the “Bipolar Disorder” diagnosis we considered Bipolar Type 1 and Type 2 disorders; for the “Psychotic Disorder” diagnosis, mood disorder with psychotic features and any lifetime major depressive, manic or hypomanic episodes were excluded. Finally, with the diagnosis of “Unipolar Depression” only recurrent major depressive episodes were included. According to MINI only major psychiatric diagnoses were assessed, with the exclusion of Axis II personality disorders.

#### Ethnicity

Given the variety of ethnicity/race definitions, three broad ethnicity major groups were formed to ensure sufficient numbers of participants for analysis: *Aboriginal, White, and Other* (Asian, Black, Latin-American, Indian-Caribbean, Middle East, Mixed, Others) groups.

### Data Analysis

Participants were grouped and compared using demographic data and psychiatric diagnosis with and without ASD using χ^2^ test and Student's *t*-test as appropriate. Multivariate logistic regression analysis was used to calculate odds ratios for the factors associated with the presence of ASD, on demographic variables and mental illness diagnosis. All analyses were performed using the software Statistical Package for the Social Sciences (SPSS) Version 20.0. Statistical significance set at *p* < 0.05.

## Results

Inclusion criteria yielded 1,585 participants with a mean age of 40 ± 10 (range: 18–74). A total of 1,067 (67.3%) were male; 896 (56.5%) had <9 years of formal education; 1,516 (95.6%) lived alone; 1,505 (95.0%) were unemployed; 790 (49.8%) were White and 382 (24.1%) were Aboriginal; and 1,318 (83.1%) were born in North, Central and South America. Of the 1,585 subjects, 644 had a history of psychotic disorder (40.6%); 500 had a history of bipolar disorder (31.5%) and 441 had a history of unipolar depression (27.8%). 746 (47.1%) and 579 (36.5%) individuals were affected by substance and alcohol dependence, respectively. Administration of the MINI identified 977 participants with alcohol and/or substance dependence (ASD) and 608 without alcohol and/or substance dependence (not-ASD). Table 1 shows sample demographic characteristics and mental illness diagnosis. No differences in living situation (alone or otherwise), sex and occupation (employed or unemployed) were found among groups. ASD homeless are more frequently younger, with low education, Aboriginal and born in North, Central and South America (The Americas). The proportion of participants with ASD was highest in the Western Canada (Winnipeg and Vancouver), and significantly lower in Central (Toronto, Montreal) and Eastern Canada (Monckton). Regarding mental illness diagnosis, ASD was significantly more prevalent among people affected by bipolar disorder and unipolar depression and less prevalent among people with a psychotic disorder.

**Table 1:** Sample demographic characteristics and mental illness diagnosis of 1,585 homeless with and without ASD (only homeless with a psychiatric disorder are included).

	**ASD** ***N* = 977**	**Not-ASD** ***N* = 608**		
	**M ± sd** **(range)**	**M ± sd** **(range)**	***T***	***p***
Age	39.01 ± 10 (18–66)	43.07 ± 11.8 (19–74)	7.00	<0.001
	%	%	Chi	p
Sex (male)	67.6^a^	66.9^a^	0.06	0.826
Low educational level (<9 years)	63.1^a^	46.1^b^	44.06	<0.001
Living alone	95.8^a^	96.5^a^	0.56	0.507
Unemployed	94.9^a^	95.4^a^	0.20	0.721
Place of birth (North, Central and South America)	97.6^a^	90.5^b^	34.06	<0.001
Ethnicity Aboriginal White	32.1^a^ 47.6^a^	11.2^b^ 53.5^b^		
Others	20.3^a^	35.4^b^	103.64	<0.001
Site Moncton Montreal Toronto Winnipeg	9.7^a^ 13.8^a^ 16.2^a^ 32.7^a^	7.6^a^ 21.4^b^ 30.1^b^ 17.3^b^		
Vancouver	27.6^a^	23.7^a^	83.95	<0.001
Diagnoses Bipolar Disorder Unipolar Depression	36.8^a^ 29.9^a^	23.0^b^ 24.5^b^		
Psychotic Disorders	33.3^a^	52.5^b^	60.60	<0.001

a, b *notes stand for discrimination between groups*.

Table 2 presents the results of the logistic regression analysis where substance dependence is the criterion and demographic and diagnostic information are predictors. The results show that homeless people with ASD were more likely to be Aboriginal, have bipolar disorder and (to a lesser extent) unipolar depression. Moreover, individuals with ASD were more likely to have low educational level, be born in the Americas and not live in Montreal (Central and Eastern Canada). Gender, living alone, and working situation did not have a significant association with ASD.

**Table 2:** Logistic regression: Substance Dependence is the criterion.

**Step**	**Variable**	***N***	***B***	**OR**	**95% CI**	***p***
1	Other ethnicity Aboriginal	268 379	0.89	1.00 2.43	1.53–3.84	<0.001
2	Psychotic Disorder Bipolar Disorder Unipolar Depression	506 469 402	0.84 0.56	1.00 2.33 1.75	1.75–3.10 1.30-2.35	<0.001 <0.001
3	Educational level, low (less than 9 years)	817		1.00		
	High (more than 9 years)	560	−0.52	0.59	0.46–0.75	<0.001
4	Born in North, Central and South America	1310		1.00	
	Born not in North, Central and South America	67	−1.03	0.35	0.19–0.65	<0.001
5	Vancouver center Montreal center	371 235	−0.58	1.00 0.55	0.39–0.79	<0.001
	**Variable not in the equation**					
	Gender					
	Living alone					
	Working situation					

*Basic demographic information and mental illness diagnosis are predictors*.

*The table shows the OR to be diagnosed as Dual Disorder in 1,377 Homeless. Only variable that reached the statistical significance are listed*.

*Statistics: chi square 158.13 df = 10 p < 0.001*.

## Discussion

The present study examined the comorbidity between alcohol and/or substance use and major psychiatric diagnosis, specifically bipolar disorder, unipolar depression and psychotic disorder- in a large multi-center sample of homeless people from across Canada. The primary finding is that substance dependence is associated with significantly greater odds of being diagnosed with bipolar disorder (and to a lesser extent unipolar depression) as opposed to psychotic disorder, and that comorbidity varies by educational level, city and ethnicity. Moreover, individuals of Aboriginal descent were more likely to be diagnosed with ASD, and ASD homeless were also likely to be younger, less well-educated, and born in the Americas.

Gender, living status, and employment status did not show an association with ASD, raising the question of the role of housing and employment opportunities in mediating ASD given that gender is critical in psychopathology, traumatic experience and substance abuse (32). Although there is some evidence that gender might influence the choice of substances of abuse (33), the present study found no differences in ASD which may be explained by the high prevalence of poly substance abuse among the homeless regardless of gender (34).

Ethnicity is a complex concept that takes into account not just inherited characteristics, but also other aspects such as religion, language, customs, history and the political establishment that influence the organization of medical services (35). The present study found that the odds of being affected by ASD were associated with Aboriginal ethnicity. These results are not surprising and are entirely consistent with studies of Aboriginal peoples living in urban communities (36, 37). Indeed, tobacco, illicit and prescription drug use disorders are 2–4 times more prevalent among Aboriginal peoples in North America than in the general population (38, 39). Ethnicity seems to play a role in the rate of substance use overall (40, 41) and of alcohol use in adolescents and young adults (42).

Of particular interest is the finding of significant regional differences. In general, the odds of homeless having ASD were lower in Central and Eastern Canada (Toronto, Montreal, Monckton) than in Western Canada (Winnipeg and Vancouver). These findings show that homeless populations vary meaningfully between cities, limiting the generalizability of findings across different homeless populations. Several local factors may have contributed to our results and should be investigated further (e.g., population rate, work possibilities, social programs, and health promotions, and data collection bias).

ASD is associated with significantly greater odds of being diagnosed with bipolar disorder (and to a lesser extent unipolar depression). Reasons why bipolar disorder is associated with substance use disorder can be gleaned from the extant literature. Several studies have focused on the relationship between mental illness and substance abuse (16, 18, 21–23, 43–45). Particular attention has been paid to the role of bipolar diagnosis (46, 47); more than half subjects with bipolar disorder in the ECA Survey had a alcohol or substance dependence (14) and more than one third of individuals with hypomania had a comorbid substance use (48) or substance use disorder (49). A popular explanatory construct in the relationship between substance abuse and mental illness is the “self-medication hypothesis,” as originally described by Khantzian (50), suggesting that suffering is at the heart of addictive disorders. Individuals would then use, abuse, and become dependent upon substances because they relieve states of distress and there would be a considerable degree of psychopharmacologic specificity in an individual's preferred drug. If this theory appears to be acceptable for the use of alcohol and tranquilizers upon depression and anxiety (to relieve the “moral pain” of depression and agoraphobic cues, respectively) (51), the “self-medication hypothesis” seems not to be applicable for the use of stimulants in bipolar disorder individuals (51). Indeed, bipolar patients have also been reported to increase stimulant use during mania in order to accentuate the manic high (51, 52). The extraordinary high prevalence of co-occurring stimulant use associated with bipolar disorder cannot be reduced to “self-medication hypothesis” as it was originally described and it would be better interpreted in the light of a “self-enhancement” hypothesis (50, 51, 53–55). The drive to enhance and maintain the manic state in bipolar individuals set its roots on a common background proposed for both disorders that is characterized by instability, excitement and impulsivity; in line with it a unitary perspective as been proposed for bipolar disorders and addiction (56–58). In this broad conceptualization of bipolar disorder and substance use, regarding opioids, it cannot be rule out the possibility that bipolar heroin addicts use heroin in a self-therapeutic manner. Still, any unprovable initial beneficial effect exerted by the substance on emotional instability would soon be followed by a mood-destabilizing action, which would accelerate the course of the illness and ultimately lead to a worse clinical presentation, both on the addictive and on the psychopathological plane (59). Even if the hypothesis of self-medication and self-enhancement help in elucidating the relations between specific psychiatric illness and single substance use, the presence of “bipolarity” (the bipolar spectrum) would than be the drive guiding the progression from single substance dependence to multiple-dependence and poly-addictions leading to more severe and complex clinical presentation (60).

The relation between bipolar disorder and bipolarity (considered as a spectrum) is gaining interest especially from a clinical perspective (61), and when a specific tool is used to facilitate bipolar disorder recognition the prevalence of bipolar disorder diagnosis increases compared to depression (62, 63). In fact, evidence from the Zurich Study shows that the traditional association between major depression and SUD may be misleading and based on an under diagnosis of bipolarity, because a widening of the definition shifted the comorbidity to bipolar disorder type 2, and the association with major depressive disorder dwindled to insignificance (62). To obtain a clearer understanding of the depressive phenotype, it is pivotal that we increase our detection of hypomanic symptoms so that clinicians can better distinguish bipolar disorder type 2 from unipolar depression. Diagnostic criteria sensitive to hypomanic symptoms have been identified that suggest bipolar II disorder is at least as prevalent as major depression. Moreover, the comorbidities of these illnesses are very different and alcoholism in particular appears to be a greater problem in bipolar disorder type 2 than in unipolar depression (64). These results are consistent with our findings, which show a greater odd of ADS in bipolar disorder compared to unipolar depression.

At the other side, Psychotic Disorder homeless show a lower prevalence in ASD-group compared with Bipolar Disorder ones, even if it is well-known that substances of abuse are able to provoke psychoses; the majority of these both in intoxication and in withdrawal phases (with the only exceptions of cannabis and opioids) (65). Psychosis can be “acute” consequences of substance abuse and dependence (“induced-psychosis”) (66, 67), but they can also become independent, leading to a clinical presentation of chronic psychosis (“schizophrenia”) (68). The only substances that seems not to be related to intoxication-psychosis are opioids, in fact psychosis seems to develop prior to heroin/opioid abuse in psychotic-heroin use disorder individuals (69) resembling a sort of “self-medication” (70, 71). Surely, further studies are needed to shed light on this tricky relationship.

## Limitations

Data collected are self-reported and no urinalyses were performed. We considered ASD in the previous 12 months diagnosed according to MINI. The MINI does not allow for giving the single diagnosis of either cocaine, inhalant, stimulant, opioid, tranquilizer, or hallucinogen dependence. Thus, for the aim of the present study alcohol and substance dependence were collected together. The presence of polyabuse and poly-dependence should be taken into account in this population.

## Conclusions

Our findings support an association between alcohol and/or substance dependence and bipolar disorders among people who are homeless. The strength of this relationship and the prevalence of comorbid ASD varied in our sample based on sociodemographic and regional factors, reinforcing the need to adapt clinical services to the assessed needs of local patients.

## Author Contributions

AM, KJ, and MK drafted the strategy of analysis and the present manuscript. AM, SB, and MN reviewed the literature. AM made statistical analyzed. AM, SB, KJ, MK, JS, CS, and MK critically revised the article. All authors read and approved the final manuscript.

### Conflict of Interest Statement

MK was the co-PI of At Home/Chez Soi Vancouver as funded by Health Canada-Mental Health Commission of Canada (MHCC). The remaining authors declare that the research was conducted in the absence of any commercial or financial relationships that could be construed as a potential conflict of interest.
